# Corrigendum to “Relationship between Pain Behavior and Changes in KCNA2 Expression in the Dorsal Root Ganglia of Rats with Osteoarthritis”

**DOI:** 10.1155/2021/2890243

**Published:** 2021-03-19

**Authors:** Qihong Zhao, Lei Fan, Jiafeng Wang, Xiaoming Deng

**Affiliations:** ^1^Department of Anesthesiology, The Second Affiliated Hospital, Bengbu Medical College, Bengbu, Anhui, China; ^2^Department of Anesthesiology and Intensive Care, The First Affiliated Hospital, The Naval Military Medical University (The Second Military Medical University), Shanghai, China; ^3^Department of Orthopedics, The Third Affiliated Hospital, Sun Yat-sen University, Guangzhou, Guangdong, China

In the article titled “Relationship between Pain Behavior and Changes in KCNA2 Expression in the Dorsal Root Ganglia of Rats with Osteoarthritis” [1], the authors have identified an error with the data in Figure 1 and the corresponding section in the results. The reason for this error is that one-way ANOVA followed by LSD test was wrongly used for intra-group and inter-group comparison. After revision, Multivariate Analysis was used for analyzing data of PWMT and Simple Effect Analysis was used for analyzing data of PWTL, according to results of Mauchly's Test of Sphericity and Tests of Within-Subjects Effects.

Figure 1 should be updated as follows:

Section 3.1 of the results section should be corrected as follows.

 
*3.1. Changes in Pain Behavior in the Three Groups of Rats*
  Compared with baseline, PWMT (at two, four, and six weeks) and PWTL (at one, two, four, and six weeks) were significantly decreased in the OA group (*P* < 0.05 or 0.01) after intra-articular injection; PWMT and PWTL were lowest at four weeks (*P* < 0.01). There was no significant difference in PWMT and PWTL between four and six weeks after OA model establishment (*P* > 0.05). Compared with group C, at two, four, and six weeks, PWMT and PWTL were significantly decreased in the OA group (*P* < 0.05 or 0.01) (Figure 1).

These errors were due to a mistake in the processing of the data during the statistical analysis; however, the authors confirm that this does not affect the conclusions of the article.

## Figures and Tables

**Figure 1:**
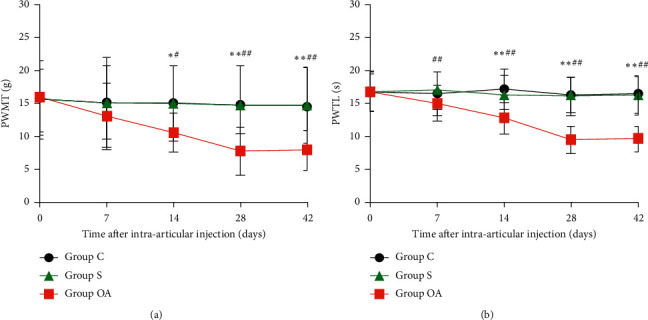
Comparison of PWMT (a) and PWTL (b) in the three groups of rats at each time point. Each symbol represents mean ± SD (standard deviation), n = 12 per group; ^∗^*P* < 0.05, ^∗∗^*P* < 0.01 vs. group C; ^#^*P* < 0.05, ^##^*P* < 0.01 vs. baseline in the OA group. Repeated measures ANOVA: (a) multiple groups; (b) multiple groups × time.

## References

[1] Zhao Q., Fan L., Wang J., Deng X. (2020). Relationship between pain behavior and changes in KCNA2 expression in the dorsal root ganglia of rats with osteoarthritis. *Pain Research and Management*.

